# Langerhans Cell Histiocytosis: A Population-based Study of Anatomical Distribution and Treatment Patterns

**DOI:** 10.1016/j.jbo.2022.100454

**Published:** 2022-09-27

**Authors:** Xianglin Hu, Ilia N. Buhtoiarov, Chunmeng Wang, Zhengwang Sun, Qinyuan Zhu, Wending Huang, Wangjun Yan, Yangbai Sun

**Affiliations:** a Department of Musculoskeletal Surgery, Fudan University Shanghai Cancer Center, Department of Oncology, Shanghai Medical College, Fudan University, Shanghai, China; b Department of Pediatric Hematology/Oncology and Bone Marrow Transplantation, Cleveland Clinic Children's Hospital, Cleveland, OH, USA; c Department of Dermatology, Huashan Hospital, Fudan University, Shanghai, China

**Keywords:** Langerhans cell histiocytosis, Treatment, Surgery, Chemotherapy, Racial disparity, Surveillance, Epidemiology and end results

## Abstract

**Background:**

Langerhans cell histiocytosis (LCH) is a rare monoclonal histiocytic neoplasm. Little is known about clinical factors associated with LCH single- vs multi-system involvement at the time of diagnosis.

**Methods:**

Data on 1549 LCH patients diagnosed between years 2010 and 2018 were extracted from the Surveillance, Epidemiology and End Results Program. Patterns of single- vs multisystem involvement were examined using multivariable logistic regression analysis. Odd ratio (OR) and 95% confidence interval (CI) were reported.

**Results:**

968 children and adolescents (0–19 years; median: 4 years) and 581 adults (≥20 years; median: 49 years) were included in the analysis. Multi-system LCH was reported for 30.9 % patients. Bone marrow (BM) (OR = 3.776; 95 %CI = 1.939–7.351; P < 0.001) and lymph node (LN) (OR = 3.274; 95 %CI = 1.443–7.427; P = 0.005) involvement were most commonly associated with multi-system LCH at the time of diagnosis; similar pattern was also observed in adult patients (OR = 17.780; 95 %CI = 6.469–48.867; P < 0.001 for BM LCH; and OR = 5.156; 95 %CI = 2.131–12.471; P < 0.001 for LN LCH). Among pediatric patients, craniofacial osseous LCH was more likely to be treated with surgery (OR = 2.822; 95 %CI = 1.199–6.639; P = 0.018) compared to skeletal lesions in other sites, whereas vertebral body LCH was less likely to be treated with surgery (OR = 0.175; 95 %CI = 0.058–0.527; P = 0.002). In pediatric patients with bone LCH, the non-white patients were less likely to be treated surgically compared to the white patients (OR = 0.470; 95 %CI = 0.272–0.812; P = 0.007).

**Conclusions:**

BM and LN LCH are associated with the highest risks of multi-system disease, which may require active surveillance. Furthermore, active attempts are needed to mitigate the racial disparity in surgery utilization in pediatric patients with skeletal LCH.

## Highlights

• LCH in bone marrow and lymph node are more likely to have multi-system involvement compared to LHC in other sites.
• Craniofacial osseous LCH is more likely to be treated with surgery, vertebral LCH is less likely to be treated with surgery.
• A racial disparity in surgery utilization is identified in pediatric patients with bone LCH.

## Graphical abstract

**Figure:**
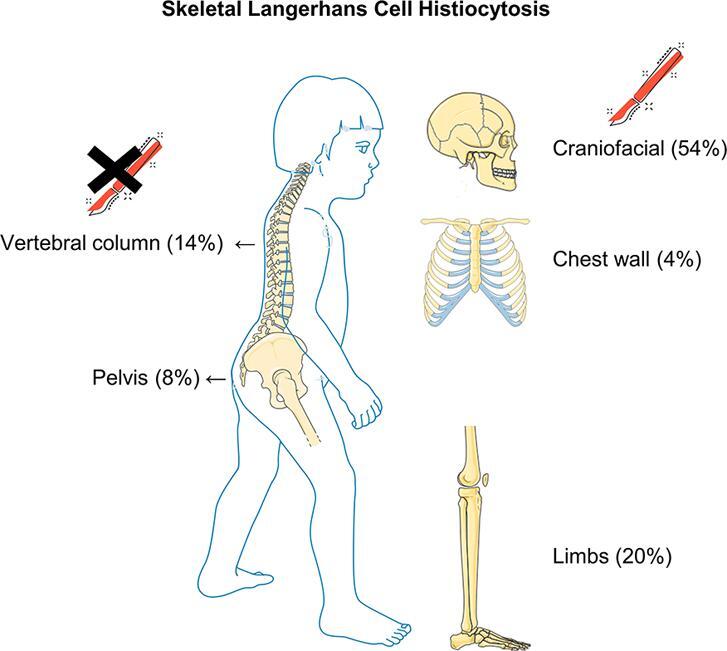

## 1 Introduction

LCH is a rare neoplastic disease characterized by clonal expansion of CD1a^+^/CD207^+^ histiocytes, also known as Langerhans cells, in the background of cellular inflammatory infiltrate [1], [2]. The yearly incidence of LCH varies by age, and is approximately 4.46 per million children and 1.06 per million adults [3]. While the etiology of LCH remains to be elucidated, there are some recognized predisposition risk factors such as family history of LCH, cancer, or thyroid disease; child born to a parent with history of occupational exposure to certain organic solvents; metal, granite or wood dust; tobacco smoking; and infections in the neonatal period [4]. Under normal physiological circumstances, Langerhans cells orchestrate immune cells cross-talk. However, upon acquired somatic BRAF^V600E^ mutation, or aberrant activation of the MAPK/ERK signaling pathway, Langerhans cells abnormally proliferate and activate surrounding reactive lymphocytes, eosinophils and neutrophils, resulting in localized or diffuse tissue distruction [5], [6], [7].

Solitary LCH lesions, single or multiple, may form in any organ; several body systems may be affected at the same time, too. Bone, skin, bone marrow (BM) and lymph node (LN) are the most common organ systems involved in pediatric patients, while lung, bone, skin and BM are the commonest involved organ systems in adult patients [2]. The clinical manifestations are highly heterogeneous depending on anatomical sites. About 70 % of LCH cases are confined to the single-organ system at the time of diagnosis. However, some patients develop multi-system LCH, often with protracted and debilitating clinical course [8]. Medical management of patients with LCH is quite heterogeneous, and includes close observation, surgery, radiotherapy and pharmacological interventions: systemic and topical chemotherapy, glucocorticoids, non-steroidal anti-inflammatory drugs such as indomethacin, and signaling pathway inhibitors [9]. There recently have been a number of comprehensive reports summarizing results of multicenter clinical trials addressing discrete research questions [10], [11], [12], [13]. While the results of these ground-breaking clinical trials have changed the treatment paradigms, the patient cohort composition in these trials may not necessarily directly translatable into the real world practice patterns. The Surveillance, Epidemiology and End Results (SEER) is the United States of America-based nationwide program, which prospectively collects data on diagnosis and treatment of various types of cancer [14]. In this study, using the SEER-based data, we aimed to explore the associations of anatomical site distribution with multisystem involvement risk and treatment pattern in pediatric and adult patients with LCH.

## 2 Methods

### 2.1 Database and patient

**Fig. 1:**
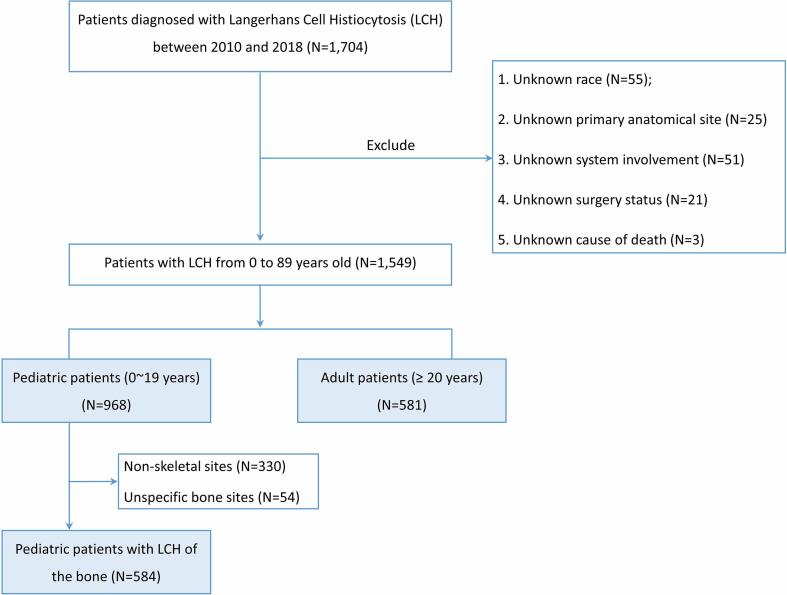
Flowchart showing patient’s selection.

In this population-based cohort study, we retrieved data from the SEER program using SEER*Stat software (8.3.9.2) (https://seer.cancer.gov/). Patients diagnosed with LCH between 2010 and 2018 were searched for based on “ICD-O-3 histology/behavior, malignant “9751/3” code. Initially, 1704 patients were identified. Patients of unknown race (n = 55), unknown primary site (n = 25), unknown system involvement information (n = 51), unknown surgery status of primary site (n = 21) and unknown cause of death (n = 3) were excluded from subsequent analysis. Ultimately, data on 1549 patients were analyzed; 968 were pediatric patients (0–19 years) and 581 were adult patients (≥20 years). The patient inclusion flowchart is displayed in Fig. 1.

### 2.2 Clinical variables

Data on the patients’ age at diagnosis; sex; race (white vs non-white as defined in the SEER database); primary site; diagnostic confirmation approach; surgery status for primary site; type of therapy; survival time, and cause of death were extracted. Primary sites of the bone were further divided into craniofacial (C41.0–1, including mandible), limb (C40.0–3), vertebral (C41.2), pelvic (C41.4) and chest wall (C41.3). Body system involvement at the diagnosis was extracted using the field “combined summary stage”, where “localized” was considered as the single-system involvement, while “distant” as the multi-system involvement. Disease specific survival (DSS) and overall survival (OS) were also determined.

### 2.3 Statistical analysis

Age was expressed by median with quartiles and dichotomized based on the median. Categorical variables, such as sex, race and etc., were expressed by number with percentage, and examined by the Chi-square test. Multivariable logistic regression model was used to determine the associations of the anatomical body site with the outcomes. Odd ratio (OR) and 95 % confidence interval (95 %CI) were calculated. All statistical analyses were conducted using SPSS 26.0 software. A two-sided *P* < 0.05 was considered as statistically significant.

## 3 Results

### 3.1 Patient’s characteristics

Pediatric patients’ median age was 4 (1–10) years. Of them, 62.2 % were males; 16.7 % were non-white; 86.9 % had the diagnosis confirmed histologically; 30.9 % had multi-system involvement at initial diagnosis; 36.1 % underwent primary lesion surgery, 1.2 % underwent radiotherapy, and 47.7 % underwent chemotherapy (Table 1).

**Table 1:** Demographics and clinical characteristics of patients with LCH according to age and anatomical distribution.

Characteristics	Pediatric patients (0–19 years)							Adult patients (≥20 years)							
	All sites (n = 968)	Bone (n = 638)	Skin (n = 132)	BM (n = 68)	LN (n = 35)	Others (n = 95)	*P*	All sites (n = 581)	Lung (n = 209)	Bone (n = 163)	Skin (n = 61)	BM (n = 37)	LN (n = 36)	Others (n = 75)	*P*
Age, years															
Median (quartiles)	4 (1–10)	6 (2–10)	0 (0–1)	3 (1–8)	1 (0–6)	5 (2–11)		49 (35–60)	54 (45–61.5)	38 (30–52)	55 (39.5–67)	50 (38.5–71)	53 (37.5–68)	45 (28–58)	
0 ∼ 4	500 (51.7)	274 (42.9)	118 (89.4)	40 (58.8)	25 (71.4)	43 (45.3)	<0.001*	299 (51.5)	80 (38.3)	116 (71.2)	25 (41.0)	17 (45.9)	16 (44.4)	45 (60.0)	<0.001*
5 ∼ 19	468 (48.3)	364 (57.1)	14 (10.6)	28 (41.2)	10 (28.6)	52 (54.7)		282 (48.5)	129 (61.7)	47 (28.8)	36 (59.0)	20 (54.1)	20 (55.6)	30 (40.0)	

Sex															
Female	366 (37.8)	229 (35.9)	61 (46.2)	26 (38.2)	12 (34.3)	38 (40.0)	0.254	316 (54.4)	128 (61.2)	83 (50.9)	32 (52.5)	16 (43.2)	15 (41.7)	42 (56.0)	0.104
Male	602 (62.2)	409 (64.1)	71 (53.8)	42 (61.8)	23 (65.7)	57 (60.0)		265 (45.6)	81 (38.8)	80 (49.1)	29 (47.5)	21 (56.8)	21 (58.3)	33 (44.0)	
Race															
White	806 (83.3)	546 (85.6)	104 (78.8)	55 (80.9)	27 (77.1)	74 (77.9)	0.110	483 (83.1)	167 (79.9)	138 (84.7)	54 (88.5)	30 (81.1)	31 (86.1)	63 (84.0)	0.628
Non-white	162 (16.7)	92 (14.4)	28 (21.2)	13 (19.1)	8 (22.9)	21 (22.1)		98 (16.9)	42 (20.1)	25 (15.3)	7 (11.5)	7 (18.9)	5 (13.9)	12 (16.0)	

Diagnostic basis															
Positive histology	841 (86.9)	555 (87.0)	122 (92.4)	61 (89.7)	25 (71.4)	78 (82.1)	0.010*	473 (81.4)	162 (77.5)	142 (87.1)	51 (83.6)	31 (83.8)	22 (61.1)	65 (86.7)	0.004*
Others	127 (13.1)	83 (13.0)	10 (7.6)	7 (10.3)	10 (28.6)	17 (17.9)		108 (18.6)	47 (22.5)	21 (12.9)	10 (16.4)	6 (16.2)	14 (38.9)	10 (13.3)	

System involvement at the diagnosis															
Single system	669 (69.1)	484 (75.9)	83 (62.9)	24 (35.3)	13 (37.1)	65 (68.4)	<0.001*	415 (71.4)	169 (80.9)	122 (74.8)	40 (65.6)	7 (18.9)	16 (44.4)	61 (81.3)	<0.001*
Multi-system	299 (30.9)	154 (24.1)	49 (37.1)	44 (64.7)	22 (62.9)	30 (31.6)		166 (28.6)	40 (19.1)	41 (25.2)	21 (34.4)	30 (81.1)	20 (55.6)	14 (18.7)	
Surgery															
No	619 (63.9)	362 (56.7)	105 (79.5)	68 (1 0 0)	27 (77.1)	57 (60)	<0.001*	338 (58.2)	104 (49.8)	79 (48.5)	42 (68.9)	37 (1 0 0)	27 (75.0)	49 (65.3)	<0.001*
Yes	349 (36.1)	276 (43.3)	27 (20.5)	0	8 (22.9)	38 (40)		243 (41.8)	105 (50.2)	84 (51.5)	19 (31.1)	0	9 (25.0)	26 (34.7)	

Radiotherapy															
No	956 (98.8)	626 (98.1)	132 (1 0 0)	68 (1 0 0)	35 (1 0 0)	95 (1 0 0)	0.039*	535 (92.1)	209 (1 0 0)	129 (79.1)	60 (98.4)	34 (91.9)	36 (1 0 0)	67 (89.3)	<0.001*
Yes	12 (1.2)	12 (1.9)	0	0	0	0		46 (7.9)	0	34 (20.9)	1 (1.6)	3 (8.1)	0	8 (10.7)	

Chemotherapy							<0.001*								
No	506 (52.3)	347 (54.4)	91 (68.9)	21 (30.9)	11 (31.4)	36 (37.9)		466 (80.2)	194 (92.8)	125 (76.7)	42 (68.9)	30 (81.1)	21 (58.3)	54 (72.0)	<0.001*
Yes	462 (47.7)	291 (45.6)	41 (31.1)	47 (69.1)	24 (68.6)	59 (62.1)		115 (19.8)	15 (7.2)	38 (23.3)	19 (31.1)	7 (18.9)	15 (41.7)	21 (28.0)	

Abbreviations: LCH: Langerhans cell histiocytosis; BM: Bone marrow; LN: Lymph node.

In pediatric patients, bone (65.9 %), skin (13.4 %), BM (7.0 %), and LN (3.6 %) were the four most commonly involved primary sites. Patients with LCH involving BM (64.7 %) and LN (62.9 %) had considerably higher rate of multi-system disease at the time of diagnosis. A low frequency of the primary lesion surgery (excisional biopsy for diagnosis) was found for skin (20.5 %) and lymph nodes (22.9 %). Radiotherapy was only used for the skeletal sites (1.9 %). Chemotherapy was less frequently used for the bone (45.1 %) and skin (31.1 %) lesions (Table 1). During a median follow-up of 41 months (quartiles; 18–71; range; 0–107 months), only one patient died of LCH (five-year DSS: 100.0 %); fifteen patients died of all-causes (five-year OS: 98.5 %).

The adult patients’ median age was 49 years (range: 20–89 years). Of them, 45.6 % were males; 16.9 % were non-white; 81.4 % had their diagnosis conformed histologically; 28.6 % presented with multi-system involvement at initial diagnosis; 41.8 % underwent primary lesion surgery, 7.9 % underwent radiotherapy, and 19.8 % underwent chemotherapy (Table 1).

In adult patients, lung (36.0 %), bone (28.1 %), skin (10.5 %), BM (6.4 %) and LN (6.2 %) were the five commonest primary sites. LCH in BM (81.1 %) and LN (55.6 %) had a higher rate of multi-system disease at the time of diagnosis. A high rate of primary lesion diagnostic biopsy was found for lung (50.2 %) and bone (51.5 %). Radiotherapy was most frequently applied to the bone lesion (20.9 %). Chemotherapy was less commonly used for treatment of lung LCH (7.2 %) (Table 1). During a median follow-up of 37 months (quartiles: 16–63; range: 0–107 months), eighteen patients died of LCH (five-year DSS: 95.9 %); 65 patients died of all-causes (five-year OS: 87.0 %).

### 3.2 Association of anatomical sites with multi-system disease

**Fig. 2:**
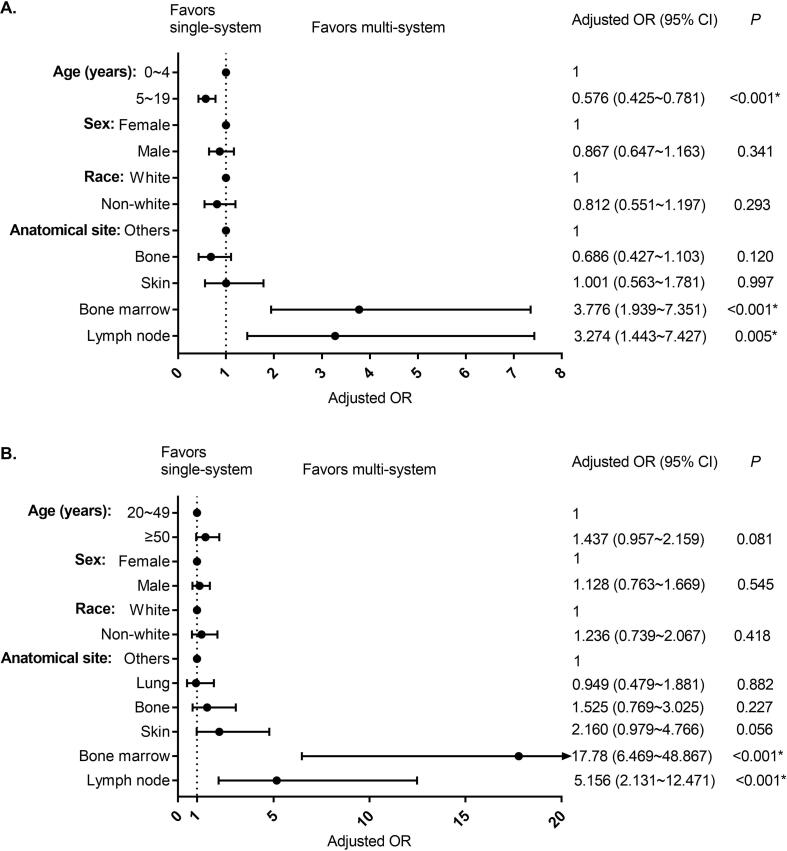
Forest plots showing factors associated with multi-system disease in pediatric (A) and adult (B) patients with LCH.

For pediatric patients, LCH in BM (OR = 3.776; 95 %CI = 1.939–7.351; P < 0.001) and LN (OR = 3.274; 95 % CI = 1.443–7.427; P = 0.005) were more likely to have multi-system involvement compared to LHC in other sites (Fig. 2**A**). Patients from 5 to 19 years of age were less likely to have multi-system involvement than patients of 0–4 years of age (OR = 0.576; 95 %CI = 0.425–0.781; P < 0.001).

Similar to results for pediatric patients, adult patients with LCH in BM (OR = 17.78; 95 %CI = 6.469–48.867; P < 0.001) and LN (OR = 5.156; 95 %CI = 2.131–12.471; P < 0.001) were also more likely to have multi-system involvement compared to adult patients with LCH in other sites (Fig. 2**B**).

### 3.3 Association of anatomical sites with treatments in pediatric patients

**Fig. 3:**
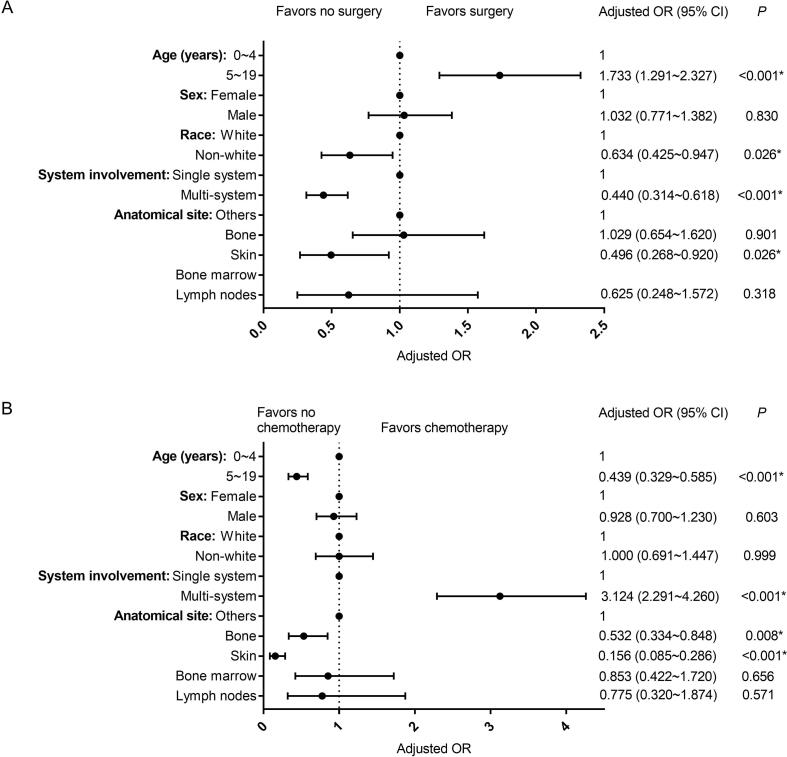
Forest plots showing factors associated with treatments in pediatric patients with LCH (0–19 years).

Pediatric patients with LCH of the bone were less likely to undergo chemotherapy compared to their counterparts with LCH of other sites (OR = 0.532; 95 %CI = 0.334–0.848; P = 0.008). The pediatric patients of 5–19 years of age were more likely to undergo surgery (OR = 1.733; 95 %CI = 1.291–2.327; P < 0.001) and less likely to undergo chemotherapy (OR = 0.439; 95 % CI = 0.329–0.585; P < 0.001) compared to those of 0–4 years of age (Fig. 3).

### 3.4 Skeletal LCH in pediatric patients

The demographic and clinical characteristics of pediatric patients with LCH affecting different skeletal anatomical sites are detailed in Table 2. The frequency of successful diagnostic biopsy with positive histological findings were high in craniofacial (88.2 %), limb (90.8 %), pelvic (88.9 %) bone LCH, and somewhat lower for lesions located in vertebral column (75.3 %). LCH of craniofacial bones was commonly treated with surgery (63.6 %), while LCH involving vertebral column bones were treated surgically rather rarely (9.9 %). Radiotherapy was more commonly used for treatment of vertebral (4.9 %) and pelvic (6.7 %) LCH, but less in craniofacial (0.6 %) and limb (1.7 %) bone LCH. Similarly, chemotherapy was a treatment of choice for LCH of bones of chest wall and limbs only in 12.0 % and 18.3 % cases, respectfully.

**Table 2:** Demographics and clinical characteristics of pediatric patients with skeletal LCH (0 ∼ 19 years).

Characteristics	All (n = 584)	Craniofacial (n = 313)	Limb (n = 120)	Vertebral (n = 81)	Pelvic (n = 45)	Chest wall (n = 25)	*P*
Age, years							
Median (quartiles)	6 (2.25 ∼ 11)	7 (3 ∼ 11.5)	5 (2 ∼ 9)	6 (3 ∼ 9)	5 (2 ∼ 10.5)	9 (4 ∼ 12.5)	
0 ∼ 4	236 (40.4)	117 (37.4)	56 (46.7)	35 (43.2)	21 (46.7)	7 (28.0)	0.220
5 ∼ 19	348 (59.6)	196 (62.6)	64 (53.3)	46 (56.8)	24 (53.3)	18 (72.0)	

Sex							
Female	207 (35.4)	111 (35.5)	40 (33.3)	34 (42.0)	15 (33.3)	7 (28.0)	0.656
Male	377 (64.6)	202 (64.5)	80 (66.7)	47 (58.0)	30 (66.7)	18 (72.0)	
Race							
White	501 (85.8)	275 (87.9)	102 (85.0)	68 (84.0)	37 (82.2)	19 (76.0)	0.470
Non-white	83 (14.2)	38 (12.1)	18 (15.0)	13 (16.0)	8 (17.8)	6 (24.0)	

Diagnostic basis							
Positive histology	507 (86.8)	276 (88.2)	109 (90.8)	61 (75.3)	40 (88.9)	21 (84.0)	0.018*
Others	77 (13.2)	37 (11.8)	11 (9.2)	20 (24.7)	5 (11.1)	4 (16.0)	

System involvement at the diagnosis							
Single system	469 (80.3)	246 (78.6)	102 (85.0)	61 (75.3)	37 (82.2)	23 (92.0)	0.177
Multi-system	115 (19.7)	67 (21.4)	18 (15.0)	20 (24.7)	8 (17.8)	2 (8.0)	

Surgery							
No	315 (53.9)	114 (36.4)	80 (66.7)	73 (90.1)	33 (73.3)	15 (60.0)	<0.001*
Yes	269 (46.1)	199 (63.6)	40 (33.3)	8 (9.9)	12 (26.7)	10 (40.0)	
Radiotherapy							
No	572 (97.9)	311 (99.4)	118 (98.3)	77 (95.1)	42 (93.3)	24 (96.0)	0.038*
Yes	12 (2.1)	2 (0.6)	2 (1.7)	4 (4.9)	3 (6.7)	1 (4.0)	
Chemotherapy							<0.001*
No	340 (58.2)	160 (51.1)	98 (81.7)	31 (38.3)	29 (64.4)	22 (88.0)	
Yes	244 (41.8)	153 (48.9)	22 (18.3)	50 (61.7)	16 (35.6)	3 (12.0)	

Abbreviations: LCH: Langerhans cell histiocytosis.

LCH in different skeletal sites was associated with similar risk of multi-system presentation (all *P* > 0.05) (Supplementary Table 1). Craniofacial osseous LCH was more likely to be treated with surgery (OR = 2.822; 95 %CI = 1.199–6.639; *P* = 0.018) and chemotherapy (OR = 6.745; 95 %CI = 1.908–23.850; *P* = 0.003) compared to LCH in other bone sites. Vertebral LCH was less likely to be treated with surgery (OR = 0.175; 95 %CI = 0.058–0.527; *P* = 0.002) but more likely to be treated with chemotherapy (OR = 10.805; 95 % CI = 2.871–40.662; *P* < 0.001) compared to LCH in other bone sites.

**Fig. 4:**
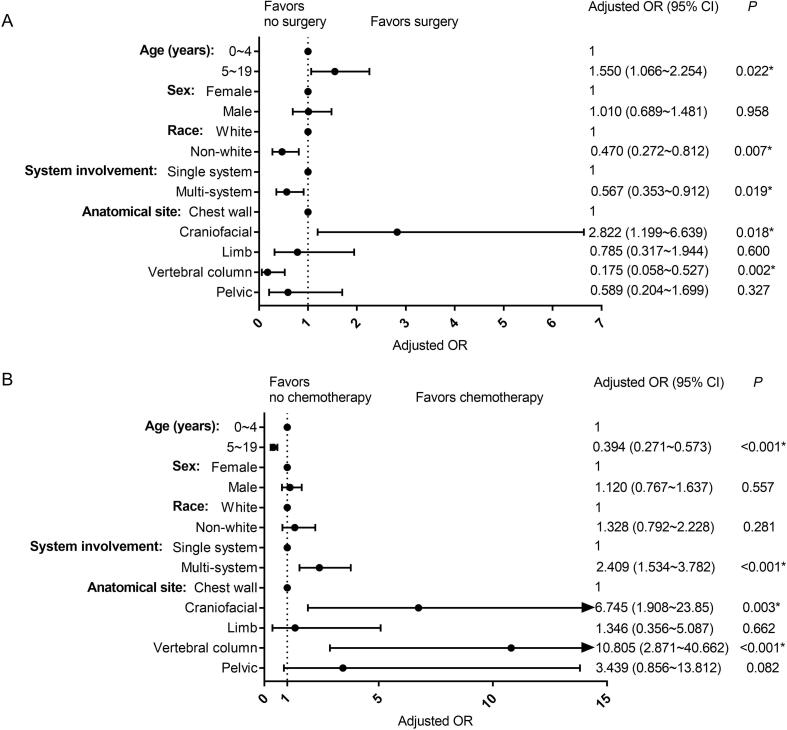
Forest plots showing factors associated with treatments in pediatric patients with skeletal LCH (0–19 years).

A racial disparity in surgery utilization was identified in pediatric patients with bone LCH: non-white patients were less likely to be treated with surgery compared to white patients (OR = 0.470; 95 %CI = 0.272–0.812; P = 0.007) (Fig. 4).

## 4 Discussion

Skeleton is the most commonly affected organ system in pediatric patients with LCH, which accounted for 65.9 % of all LCH in our data analysis. Craniofacial osseous LCH is the most common location, accounting for 53.6 % of all skeletal LCH. Our study demonstrates that craniofacial osseous LCH is treated predominantly with surgery and chemotherapy compared to skeletal LCH in other anatomical compartments. The lesion curettage is the commonest surgical method, without need of craniotomy unless the dura mater is involved. In our analysis, radiotherapy (0.6 %) was uncommonly used for craniofacial osseous LCH. Of note, recently, Hiroshima Y., et al reported that, for a skull LCH even with dura infiltration, a non-high intensity radiotherapy can rapidly shrink the lesion resulting in a complete response without local recurrence [15]. Therefore, the feasibility and safety of radiotherapy in certain clinical scenarios may need to be explored.

Osseous LCH involving spine is insidious. We previously demonstrated that, once the pathological process involves the vertebral arch or canal, patients may experience neck or back pain, limb numbness, muscle weakness and limping [16], [17]. In the last decade, 18-fluorodeoxyglucose positron emission tomography-computed tomography (^18^FDG PET-CT) plays an increasingly important role in diagnosis of LCH [18], [19], [20]. ^18^FDG PET-CT can help detect asymptomatic LCH lesions and differentiate metabolically active lesions from inactive disease [21]. In the multivariable analysis we demonstrate that osseous LCH of the spine was less likely to be treated with surgery compared to osseous LCH of other bones. Immobilization and observation are the preferred managements while surgery is only applicable for patients with evolving neurological complications [17].

In addition, our study identified a racial disparity in surgery utilization in pediatric patients with bone LCH: the non-white patients were less likely to be treated with surgery than the whites. In the United States, the racial disparity, whether or not mediated by patient’s socioeconomic status, is an important factor influencing the care and outcome of various childhood neoplasms [22], [23], [24]. Our work suggests that equitable care should be stressed in pediatric patients with osseous LCH as well. Hispanic mothers are more likely to have children with LCH compared to non-Hispanic whites (OR = 1.51; 95 %CI = 1.02–2.25); this risk increases further when both parents are Hispanic (OR = 1.80, 95 % CI = 1.13–2.87) [25]. Higher age-standardized incidence rate of LCH has also been observed for Hispanics compared to non-Hispanics (RR = 1.63; 95 % CI = 1.15–2.29) [26].

Skin is the second most commonly affected organ (13.4 % in our study) in pediatric patients with LCH. Our study demonstrated that skin LCH had the highest diagnostic rate via positive histology (92.4 %). Skin LCH has a tendency for spontaneous resolution and is less likely to be associated with multisystem LCH [27]. We demonstrated that skin LCH was less likely to be treated with surgery and chemotherapy compared to LCH of other sites. Indomethacin may be a useful option for recurrent skin LCH refractory to other frontline therapies [28].

In summary, LCH is a highly heterogeneous disease with a wide variety of clinical manifestations. In this study we demonstrate that the risks of multi-system involvement and management strategies depend upon anatomic systems involved. This work also highlights the needs to further study racial disparity in treatment option application in pediatric patients with skeletal LCH.

## 5 Ethics approval and consent to participate

Not applicable.

## 6 Availability of data and materials

All data were available from the US Surveillance, Epidemiology, and End Results Program (https://seer.cancer.gov/).

## Funding

This work was supported by the 10.13039/501100001809National Natural Science Foundation of China (#82003351; recipient: Qinyuan Zhu) and Shanghai Municipal Health Commission Research Project (# 20194Y0242; recipient: Yangbai Sun).

## Author contribution

All authors equally contributed to the analysis and writing of the manuscript.

## Declaration of Competing Interest

The authors declare that they have no known competing financial interests or personal relationships that could have appeared to influence the work reported in this paper.
