# Supplementary material for: Langerhans Cell Histiocytosis: A Population-based Study of Anatomical Distribution and Treatment Patterns
Source: J Bone Oncol. 2022 Sep 27;36:100454. doi: 10.1016/j.jbo.2022.100454 (PMC9530953; doi:10.1016/j.jbo.2022.100454)
Supplement: Supplementary data 1 [file mmc1.docx]

Supplementary table 1. Factors associated with multi-system involvement in pediatric patients with skeletal LCH (0-19 years).

| Characteristics | Adjusted OR (95% CI) | *P* |
| --- | --- | --- |
| Age |  |  |
| 0-4 | 1 |  |
| 5-19 | 0.601 (0.396-0.911) | 0.017^*^ |
| Sex |  |  |
| Female | 1 |  |
| Male | 0.751 (0.492-1.146) | 0.184 |
| Race |  |  |
| White | 1 |  |
| Non-white | 1.030 (0.571-1.857) | 0.923 |
| Site |  |  |
| Chest wall | 1 |  |
| Craniofacial | 2.956 (0.675-12.952) | 0.150 |
| Limb | 1.821 (0.391-8.479) | 0.445 |
| Vertebral | 3.380 (0.725-15.758) | 0.121 |
| Pelvic | 2.249 (0.435-11.626) | 0.333 |

Abbreviations: LCH: Langerhans cell histiocytosis; OR: odds ratio; CI: confidence interval.
